# Modeling the Inactivation of Intestinal Pathogenic *Escherichia coli* O157:H7 and Uropathogenic *E. coli* in Ground Chicken by High Pressure Processing and Thymol

**DOI:** 10.3389/fmicb.2016.00920

**Published:** 2016-06-14

**Authors:** Shih-Yung Chien, Shiowshuh Sheen, Christopher H. Sommers, Lee-Yan Sheen

**Affiliations:** ^1^Institute of Food Science and Technology, National Taiwan UniversityTaipei, Taiwan; ^2^United States Department of Agriculture, Eastern Regional Research Center, Agricultural Research ServiceWyndmoor, PA USA

**Keywords:** modeling, high pressure processing, thymol, *E. coli* O157:H7, uropathogenic *E. coli*, ground chicken

## Abstract

Disease causing *Escherichia coli* commonly found in meat and poultry include intestinal pathogenic *E. coli* (iPEC) as well as extraintestinal types such as the Uropathogenic *E. coli* (UPEC). In this study we compared the resistance of iPEC (O157:H7) to UPEC in chicken meat using High Pressure Processing (HPP) in with (the hurdle concept) and without thymol essential oil as a sensitizer. UPEC was found slightly more resistant than *E. coli* O157:H7 (iPEC O157:H7) at 450 and 500 MPa. A central composite experimental design was used to evaluate the effect of pressure (300–400 MPa), thymol concentration (100–200 ppm), and pressure-holding time (10–20 min) on the inactivation of iPEC O157:H7 and UPEC in ground chicken. The hurdle approach reduced the high pressure levels and thymol doses imposed on the food matrices and potentially decreased food quality damaged after treatment. The quadratic equations were developed to predict the impact (lethality) on iPEC O157:H7 (*R*^2^ = 0.94) and UPEC (*R*^2^ = 0.98), as well as dimensionless non-linear models [Pr > *F* (<0.0001)]. Both linear and non-linear models were validated with data obtained from separated experiment points. All models may predict the inactivation/lethality within the same order of accuracy. However, the dimensionless non-linear models showed potential applications with parameters outside the central composite design ranges. The results provide useful information of both iPEC O157:H7 and UPEC in regard to how they may survive HPP in the presence or absence of thymol. The models may further assist regulatory agencies and food industry to assess the potential risk of iPEC O157:H7 and UPEC in ground chicken.

## Introduction

While most *Escherichia coli* are harmless, some are considered pathogenic for human beings that include both intestinal pathogenic *E. coli* (iPEC) as well as extraintestinal pathogenic *E. coli* (ExPEC). *Escherichia coli* O157:H7 are Shiga toxin-producing *E. coli* (STEC) are one type of iPEC which are common contaminants in meat and poultry (Bryan et al., 2015). Between 2000 and 2010, there had 5688 cases of O157:H7 STEC infections reported by FoodNet. Many illness outbreaks due to STEC were reported including recent ones at retail restaurants and wholesale outlets (chicken salad) [Centers for Disease Control and Prevention (CDC), 2015]. Magwedere et al. (2013) reported that retail ground meat samples, 7 out of 16 ground chicken samples were tested positive of O157:H7, purchased at grocery stores, local farmers' markets, and online vendors. ExPEC include the uropathogenic *E. coli* (UPEC) which cause urinary tract infections, cystitis, and kidney infections, primarily in women (Minardi et al., 2011). UPEC are common contaminants in poultry meat and other foods and cause infection after colonization of the gastrointestinal tract followed by accidental transfer of UPEC contaminated feces from the anus to the urethra (Jakobsen et al., 2010; Markland et al., 2015; Mitchell et al., 2015; Müller et al., 2016). While STEC are responsible for ca. 300 deaths in the US annually, the ExPEC are responsible for ca. 26,000, although the percentage which could be attributed to contaminated food is currently unknown as there may be multiple routes for infection of humans by the ExPEC (Scallan et al., 2011; Nordstom et al., 2013; Singer, 2015).

Many essential oils exhibit antimicrobial activity against a range of bacteria, yeast and molds, and may be useful for improving the safety and shelf-life of foods. Several comprehensive reviews of the applications using antimicrobial compounds in food systems were available from Calo et al. (2015) and Lucera et al. (2012). Thymol, a monoterpene phenol, has been classified as generally recognized as safe (GRAS) by the U.S. Food and Drug Administration (Johny et al., 2010). The antimicrobial effect of thymol might be due to the perturbation in the lipid fractions of bacterial plasma membranes by hydrogen bonding, rendering the membranes and mitochondria more permeable and disintegrating the outer cell membrane (Trombetta et al., 2005; Di Pasqua et al., 2010; Marchese et al., 2016). It has been widely used in the microbial safety enhancement against a variety of pathogens, including *E. coli* O157:H7 (Di Pasqua et al., 2007). High pressure processing (HPP) is an effective non-thermal technology to reduce or eliminate foodborne illness risks. HPP can inactivate pathogenic microorganisms in some foods with minimal effects on the quality, for instance, colors, flavors, nutritional values, and sensory properties (Hendrickx et al., 1998; San Martín et al., 2002; Olsen et al., 2010). The applications of HPP, as an emerging technology in food processing, have steadily increased in the past several years and have received particular attention as economically and technologically more feasible in mass production scale (Patterson, 2005). The HPP impact on cell survival was discussed by Hsu et al. (2015) including several mechanisms to damage normal cell functions. Gänzle and Liu (2015) reviewed and reported the mechanisms of pressure-mediated cell death and injury in *E. coli* from fundamental to food applications which provided useful and up-to-date information. Geroget et al. (2015) reviewed the HPP inactivation mechanisms in complex food matrices including low water activity. The HPP at 400–600 MPa is effective in controlling most major foodborne pathogens (*E. coli* O157:H7, *Salmonella* spp. etc.) present in various meat products such as ground beef and ground chicken (Hsu et al., 2015; Sheen et al., 2015a,b) but caused detrimental changes in food quality.

A “hurdle” concept is combining two or more positively impacting factors to achieve an objective (e.g., inactivation) in which less quantity or lower level of each factor may be required when compared, to attaining similar results, with those using individual factor alone. Therefore, the negative impact from each factor on food quality can be much reduced. The HPP has been demonstrated an effective means to inactivate or eliminate foodborne pathogens in foods. However, a high pressure level (e.g., ≥450 MPa) may induce significant food quality deterioration. The antimicrobial dose applied in real foods was typically higher compared to that in culture media (unpublished data) to achieve same inactivation level. A high dose of antimicrobial may cause unpleasant flavor or color changes and the processed foods become unacceptable in the consumer market. Based on the hurdle concept, each kind of treatment can be used in combination with other disinfection strategies to potentiate microbial lethality (Leistner and Gorris, 1995; Chen and Jiang, 2014). Therefore, a hurdle approach may be considered in combining HPP and proper antimicrobial to lower the imposed high pressure level and reduce food quality damage and optimally achieve microbial food safety enhancement.

Using mathematical modeling to predict the HPP lethality in combination with antimicrobial and/or other hurdles may assist in assessing the risk of foodborne pathogens in finished products. Reliable mathematical models provide advantages including to estimate the results (or responses) without performing the experiment and to facilitate the scale-up, and/or process optimization. A central composite design with three parameters (i.e., HPP pressure, antimicrobial dose, and process time) for linear modeling and dimensionless non-linear modeling were used to develop proper models to predict the microbial survival potential (i.e., inactivation or lethality).

The CDC has recommended foods treated with intervention technologies such as HPP for “at risk” persons such as those with underlying medical conditions which may make them more susceptible to infection by foodborne pathogens such as the STEC or UPEC (Zink, 1997). The objective of this research was to improve the efficacy of HPP by using it in combination with thymol essential oil in a hurdle approach. In this study we report the development and validation of regression models for the inactivation of iPEC O157:H7 and UPEC in chicken meat with by combining HPP and thymol for ground chicken. The HPP resistance of iPEC O157:H7 and UPEC were also compared and reported.

## Materials and methods

### Ground chicken sample preparation

Ground chicken (ca. 95% lean, 5% fat content) purchased at a local wholesaler (Lansdale, PA) was delivered to lab in a cooler and evenly portioned into 90 g samples in polynylon pouches (Uline, Inc., Philadelphia, PA), vacuum sealed to 50 millibars using a Multi-Vac A300 packager (Multi-Vac Inc., Kansas City, MO) and then frozen (−20°C). The ground chicken was later gamma irradiated (Cs-137, 0.070 kGy/min, −20°C, Lockheed Georgia, Marietta, GA) to a dose of ca. 5 kGy which inactivated any contaminating *E. coli* in survival study. The irradiated and non-irradiated ground chicken under HPP stress showed similar lethality in conditions tested, e.g., the plate count difference was non-significant (*P* > 0.05) indicating the HPP may effectively inactivate the non-pathogenic *E. coli* strains existed in the non-treated ground chicken. The ground chicken was then maintained at −20°C. Ground chicken was thawed overnight in a refrigerator (4°C) prior to experiment procedures.

### *E. coli* cultures and cocktail preparation

iPEC O157:H7 C9490, 59762, and 59768 (isolates involved in food outbreaks including meats), and UPEC 700336, 700414, and 700415 (isolates from women with UTI) (Sommers et al., 2016) were obtained from the American Type Culture Collection (Manassas Virginia). Each *E. coli* isolate was propagated on Sorbitol MacConkey agar (BD/Difco) and stored at 4°C. Twenty-four hours before the experiment, a loopful of each strain was individually transferred to 25 ml Tryptic Soy Broth (TSB, BD/Difco) and held at 37°C in an orbital shaker (Model G34, New Brunswick Scientific, Edison, NJ) at 150 rpm for approximately 20 h. Each culture was then harvested by centrifugation, 2400 × g for 15 min at 4°C, (Model Z-206A, Hermle Labor-technik, Germany), and re-suspended in 25 ml 0.1% sterile peptone water (SPW, BD/Difco). A working cocktail was formed by combining and mixing the individually washed STEC or UPEC cultures. Every culture contained an approximate cell population of 10^8−9^ CFU/ml (colony forming unit per ml). Fresh culture cocktails were prepared for each experiment (National Advisory Committee on Microbiological Criteria for Foods (NACMCF), 2006).

### High pressure processing (HPP) treatments

HPP was performed in a laboratory scale pressure unit (Mini Food lab FPG5620, Stansted Fluid Power Ltd., Essex, UK), comprised of a double-jacketed thick-wall stainless steel cylinder (approximate volume of 0.3 L) having an internal stainless steel sample holder of 25.4 × 254 mm (diameter × length). The thick-wall cylinder was maintained at a set-point temperature in which heat transfer fluid continuously circulated from a refrigerated liquid chiller (Proline RP 855, Lauda, Germany). The refrigerated chiller was set at 4°C which indirectly cooled the pressure transmitting medium (a mixture of ethanol and castor oil, 80/20% weight basis). The pressure come-up rate was 100 MPa per 15 s (or 6.67 MPa/s) and the release rate was 100 MPa per 9 s (or 11.11 MPa/s; Hsu et al., 2015). This temperature set-up ensured that foods in the pressure chamber were maintained at <40°C during the HPP test and eliminated the potential for thermal lethality.

### Meat sample preparations for HPP treatment (without thymol)

Thawed ground chicken (5 g) was aliquoted into 2 oz Nasco Co. (Ft. Atkinson, WI) Whirl-Pak bags, inoculated with 0.5 ml of cocktail, then mixed manually for 30 s, and sealed to 50 millibars using the Multi-Vac A300 Packager. Samples receiving the same treatment were then packed and sealed in a polynylon bag (Uline, Inc., Philadelphia, PA) as a secondary barrier prior to HPP treatment. The samples were stored at 4°C while awaiting HPP treatment. For HPP treatment (without thymol, **Table 2**), each high pressure level was repeated in triplicate randomly and two samples were tested for each run.

### Thymol solution preparation and meat samples with thymol for HPP

Thymol (99.5% purity, Sigma-Aldrich, Tulsa, OK) was purchased and kept in dark/cool area. A stock solution of 5% thymol (or 50,000 ppm, w/v; solubility 50 mg/ml ethanol) was prepared by dissolving 5 g of thymol in 100.0 ml of ethanol (200 proof, KOPTEC, King of Prussia, PA) in a volumetric flask. The stock solution was freshly prepared weekly and stored in the dark (4°C). The similar procedures for sample preparation without thymol were applied; then, a proper amount of thymol solution was added and mixed well to attain the targeted thymol ppm per each central composition design point requirement (**Table 3**). For example, 0.01 ml of 5% thymol solution added to 5 g ground meat resulted in 100 ppm thymol in ground chicken.

### Central composite design (CCD)

When multiple parameters (≥3) were involved in modeling task, an experimental design typically needed to reduce the parameter combination number for experiment to be executed. A proper experimental design can save time, cost and enhance the accuracy of developed model. There were three parameters in the current case; a CCD is more efficient than a full factorial design where each parameter associated with three levels. The CCD design further expanded the parameter range (to ±α level) and could benefit the model applications in real foods.

Therefore, the CCD with three independent factors was used to study the inactivation of iPEC O157:H7 and UPEC on ground chicken. The factors investigated were pressure, thymol concentration, and pressure-holding time. Each factor at five coded levels (e.g., −1.682, −1.0, 0.0, +1.0, and +1.682) was shown in Table 1 (the ±1.682 represents ±α and each level corresponding to the physical parameter level was shown). The experimental response was the number of log reduction (log CFU/g) of iPEC O157:H7 or UPEC, which was obtained, taking into account the influence of three factors. Three key factor ranges (pressure, holding time, and thymol concentration) affecting the reduction of iPEC O157:H7 and UPEC inoculated on ground chicken were determined in a preliminary study. For HPP, optimal pressure should be approximately 300–400 MPa, and holding times at 10–20 min. To select thymol concentration ranges, sensory evaluation on odor changes of ground chicken up to 200 ppm were acceptable. Thymol at 50 ppm level was found almost no effect in cell count reduction, therefore, a range of 100–200 ppm was selected. Each combination point in the CCD design (20 total points) was repeated in triplicate randomly and have two duplicated samples taken in each run.

**Table 1:** **Variables and levels used for the Central Composition Design**.

**Factor**	**Levels**				
	**−α (−1.682)**	**−1**	**0**	**+1**	**+α (1.682)**
Pressure (MPa)	265.9	300	350	400	434.1
Concentration (ppm)	65.9	100	150	200	234.1
Time (minutes)	6.59	10	15	20	23.41

### iPEC O157:H7 and UPEC enumeration

Bags containing the HPP treated and control samples were aseptically opened. Each 5 g sample was transferred to filtered stomacher bag, 45 ml of 0.1% SPW was added to each sample bag and stomached for 2 min (Model 400C, Seward, Basingstoke, UK). Following proper decimal dilutions with 0.1% SPW, 1.0 ml of diluted sample was placed on duplicate E. coli/coliform Petrifilm™ (3M Microbiology Products Co., St. Paul, MN). In a preliminary test, TSA plate counts (Tryptic Soy agar, BD/Difco) vs. Petrifilm counts were compared and a 0.5 log CFU/g differential (with TSA counts slightly higher) was observed. However, the difference was found not significant (*p* > 0.05) as determined by ANOVA. The *E. coli*/coliform Petrifilms™ were used per USDA-FSIS also uses these films for enumeration of *E. coli* in its Microbiological Laboratory Guide (USDA Food Safety and Inspection Service (FSIS), 2012). The films were maintained at room temperature for at least 2 h to allow the injured cells to recover (Huang, 2004), and then incubated at 37°C for 24 h. Colonies were counted by the 3M Petrifilm™ plate reader (Model 6499, 3M Health Care, 3M Center, St. Paul, MN) and converted to logarithms (base 10) of colony forming units per g (log_10_ CFU/g). All Petrifilms™ were then restored at 37°C and recounted at 48 h, with no difference in recovery being observed (*p* > 0.05).

### Model development and statistical analysis

Two kinds of models (i.e., linear and non-linear) were developed to describe and predict the inactivation or survival of the pathogenic *E. coli*. The non-linear models were expected to be more flexible in applications with parameters locating outside the CCD parameter ranges. The response (inactivation) was measured as the log reduction [i.e., Log (N_*o*_/N) = Log N_*o*_ − *Log N*] of iPEC O157:H7 or UPEC populations inoculated on ground chicken vs. survival counts. The data obtained from CCD points were evaluated by statistical analysis of variance using the RSREG procedure with SAS software (SAS version 9.4, SAS Institute 2.8 Inc., Cary, NC, USA). Based on the central composite experimental design, the quadric polynomial equation/model can be obtained using the general linear regression procedures. The standard procedure set the significant criteria of each parameter term and their interaction terms (to 2nd order) at 5% level (or significant at *P* < 0.05) to reach a quadric model with the best R-squared value which can be achieved by the stepwise regression option in SAS. The collected data also can be further analyzed with the non-linear regression procedures to develop the dimensionless non-linear model (Zhou et al., 2015) using the *F*-value with Pr > F (<0.001) criteria. The significant level was set at *p* < 0.05 for general statistical analysis.

### Model validation

In order to validate the adequacy of the models/equations, two experimental combinations were selected which were not on the CCD points but still within the parameter ranges. The model validation points included thymol concentration/pressure/time at 180 ppm/320 MPa/18 min and 120 ppm/390 MPa/14 min. In addition, an extra point at 300 ppm/425 MPa/30 min was used to evaluate the performance of the dimensionless non-linear model with parameter outside the CCD ranges. Theoretically, the dimensionless non-linear model may have wider parameter application ranges. Each selected testing set point was repeated three times in random.

## Results

The pressure lethality for iPEC O157:H7 and UPEC at several pressure stresses were compared to demonstrate how the pathogenic *E. coli* may behavior differently. The combination of HPP, thymol and process time showed the hurdle concept can be adopted in ground meat to enhance food safety with reduced pressure level and/or thymol dose applied. Two proposed model types were successfully developed which have similar accuracy in lethality prediction. However, the dimensionless non-linear model may be applied to cover wider parameter ranges. The regression models have no physical meaning but provide the relatively easy and convenient means for application purpose. Important findings are detailed below.

### Impact of HPP on iPEC O157:H7 and UPEC inactivation

Inactivation data for both iPEC O157:H7 and UPEC are shown in Table 2. At 300 MPa HPP and 15 min operation conditions, the ground chicken samples showed a 0.49 log and 0.41 log CFU/g reductions for iPEC O157:H7 and UPEC, respectively. The reductions of iPEC O157:H7 and UPEC at 350 MPa and 400 MPa with 15-min process time were both about 1.6 and 2.0 log CFU/g, respectively. There was no significant difference in HPP resistance of both at pressure level up to 400 MPa (*P* > 0.05, Table 2). While pressure further increased to 450 MPa and 500 MPa, the log reduction of iPEC O157:H7 were 4.0 and 7.2, respectively. However, UPEC showed a lower reduction of 3.6 (at 450 MPa) and 5.23 log (at 500 MPa) CFU/g. The statistical analysis (two-way ANOVA) indicated the UPEC was more resistant to HPP at 450 and 500 MPa than iPEC O157:H7 (*P* < 0.05, Table 2).

**Table 2:** **Inactivation of the iPEC O157:H7 and UPEC in ground chicken treated at different pressure (300–500 MPa) for 15 min**.

**Pressure (MPa)**	**iPEC O157:H7 (in log CFU/g reduction)**	**UPEC (in log CFU/g reduction)**
300	0.49 ± 0.05^a, x^	0.41 ± 0.06^a, x^
350	1.59 ± 0.07^b, x^	1.62 ± 0.05^b, x^
400	1.98 ± 0.10^c, x^	2.05 ± 0.13^c, x^
450	4.00 ± 0.03^d, x^	3.60 ± 0.06^d, y^
500	7.20 ± 0.28^e, x^	5.23 ± 0.05^e, y^

The initial inoculum counts of the iPEC O157:H7 and UPEC were 9.01 and 9.02 log CFU/g, respectively. The detection limit was 1.0 log CFU/g. Results are shown as mean ± standard deviation (N = 3, with 2 petrifilm counts per run; n = 2 × 3 random runs). Different letter (superscript) within a column (a–e) and row (x,y) indicates significant difference (p < 0.05) among value.

### HPP and thymol effects on iPEC O157:H7 and UPEC reduction on ground chicken

A control test of added ethanol (same amount with 0 ppm of thymol) in ground chicken was examined to assess the possible effect on *E. coli* reduction and there was no such impact observed. The difference of cell counts in both iPEC O157:H7 and UPEC cases were found not significant (*p* > 0.05). Table 3 shows the reductions (or lethality) in the populations of iPEC O157: H7 and UPEC, resulting from the combined treatment with pressure, thymol, and pressure-holding time. The reduction of viability expressed as log (N_0_/N), where N_0_ is the initial number of cells (inoculum level) and N the final number of survivors after HPP. The reduction of iPEC O157:H7 and UPEC showed the range from 0.94–5.16 to 0.41–4.66 log CFU/g, respectively, in the CCD. Generally speaking, when pressure level, thymol concentration and pressure-holding time increased; iPEC O157:H7 and UPEC survival decreased. It was also noticed that the reduction of UPEC is slightly lower than iPEC O157:H7 in each CCD combination indicating UPEC may be more resistant to HPP and thymol combined. The inclusion of thymol may somewhat impact the HPP sensitivity when iPEC O157:H7 and UPEC were compared. Lethality data showed the difference was significant in every combination except the case No. 10. In the present work, an inactivation >5 log of iPEC O157:H7 and UPEC on ground chicken may be achieved with those (three) parameters at their high end levels.

**Table 3:** **Inactivation of iPEC O157:H7 and UPEC on ground chicken after high pressure processing treatment with thymol according to the Central Composite Design**.

**Trail. No.**	**Pressure MPa (level)**	**Concentration ppm (level)**	**Time minute (level)**	**Inactivation (log_10_ CFU/g reduction) Log N_o_ − Log N**	
				**iPEC O157:H7**	**UPEC**
1	300 (−1)	100 (−1)	10 (−1)	0.94 ± 0.02	0.41 ± 0.02^*^
2	300 (−1)	100 (−1)	20 (+1)	3.04 ± 0.19	1.81 ± 0.13^*^
3	300 (−1)	200 (+1)	10 (−1)	1.20 ± 0.16	0.67 ± 0.01^*^
4	300 (−1)	200 (+1)	20 (+1)	3.52 ± 0.04	1.86 ± 0.15^*^
5	400 (+1)	100 (−1)	10 (−1)	2.39 ± 0.59	1.77 ± 0.06^*^
6	400 (+1)	100 (−1)	20 (+1)	3.50 ± 0.03	4.16 ± 0.18^*^
7	400 (+1)	200 (+1)	10 (−1)	2.90 ± 0.02	1.92 ± 0.15^*^
8	400 (+1)	200 (+1)	20 (+1)	5.16 ± 0.12	4.66 ± 0.26^*^
9	266 (−α)	150 (0)	15 (0)	0.97 ± 0.10	0.50 ± 0.07^*^
10	434(+α)	150 (0)	15 (0)	3.89 ± 0.24	3.87 ± 0.13
11	350 (0)	65.9 (−α)	15 (0)	2.06 ± 0.05	1.77 ± 0.11^*^
12	350 (0)	234 (+α)	15 (0)	2.89 ± 0.03	1.80 ± 0.10^*^
13	350 (0)	150 (0)	6.6 (−α)	1.69 ± 0.07	0.94 ± 0.04^*^
14	350 (0)	150 (0)	23.4 (+α)	3.89 ± 0.11	3.23 ± 0.09^*^
15	350 (0)	150 (0)	15 (0)	3.72 ± 0.04	1.82 ± 0.05^*^
16	350 (0)	150 (0)	15 (0)	3.66 ± 0.06	2.03 ± 0.33^*^
17	350 (0)	150 (0)	15 (0)	3.54 ± 0.04	2.03 ± 0.18^*^
18	350 (0)	150 (0)	15 (0)	3.58 ± 0.04	2.14 ± 0.12^*^
19	350 (0)	150 (0)	15 (0)	3.63 ± 0.05	2.08 ± 0.10^*^
20	350 (0)	150 (0)	15 (0)	3.68 ± 0.07	1.96 ± 0.04^*^

(14 design combinations + 6 center points, No. 15–20).

The initial inoculum counts of the iPEC O157:H7 and UPEC at 8.98 and 9.02 log CFU/g, respectively. The detection limit was 1.0 log CFU/g. Results shown as mean ± standard deviation (N = 3, with 2 petrifilm counts per run; n = 2 × 3 random runs).

* Significant difference between O157 and UPEC at the same trail No. (unpaired t-test; P < 0.05).

### Response surface models for iPEC O157:H7 and UPEC reductions

Regression analysis of the experimental data with ANOVA generated the following quadratic equation to calculate the lethality (cell count reduction).

(1) $$\begin{array}{ll} \text{Log}\left({\text{N}}_{\text{o}}∕\text{N}\right)=\text{Log }{\text{N}}_{\text{o}}-\text{Log N}=\text{Y}={\text{A}}_{0}+{\text{A}}_{1}\cdot \text{P}+{\text{A}}_{2}\cdot \text{C } & \;\\ +{\text{A}}_{3}\cdot \text{T}+{\text{A}}_{4}\cdot \text{P}\cdot \text{C}+{\text{A}}_{5}\cdot \text{P}\cdot \text{T}+{\text{A}}_{6}\cdot \text{C}\cdot \text{T}+{\text{A}}_{7}\cdot {\text{P}}^{2}\; & \;\\ +{\text{A}}_{8}\cdot {\text{C}}^{2}+{\text{A}}_{9}\cdot {\text{T}}^{2}\; & \; \end{array}$$

In Equation (1), Y corresponds to the log_10_ reduction of iPEC O157:H7 or UPEC populations inoculated on ground chicken. P, C, and T are pressure, thymol concentration, and pressure-holding time, respectively. A_i(0−9)_ is the regression constant for each corresponding term.

Using the PROC GLM procedure (SAS v9.4), the polynomial models were developed for inactivation of iPEC O157: H7 and UPEC are shown in the following Equations (2) and (3), respectively, which are the quadric polynomial equations based on the central composite design of three factors and three levels through the general linear regression procedures (SAS).

iPEC O157: H7 reduction (Y_1_):

(2) $$\begin{array}{ll} {\text{Log N}}_{\text{o}}-\text{Log N}={\text{Y}}_{1}=-24.42410042+0.10645094\cdot \text{P } & \;\\ +0.01909168\cdot \text{C}+0.49827209\cdot \text{T}+0.00007133\cdot \text{P}\cdot \text{C } & \;\\ -0.00052\cdot \text{P}\cdot \text{T}+0.00068\cdot \text{C}\cdot \text{T}-0.00013498\cdot {\text{P}}^{2}\; & \;\\ -0.00016396\cdot C^{2}-0.00832561\cdot {\text{T}}^{2}\; & \; \end{array}$$

UPEC reduction (Z_1_):

(3) $$\begin{array}{ll} {\text{Log N}}_{\text{o}}-\text{Log N}={\text{Z}}_{1}=3.961984676-0.031686258\cdot \text{P } & \;\\ +0.019683572\cdot \text{C}-0.372886017\cdot \text{T}+0.001271667\cdot \text{P}\cdot \text{T } & \;\\ +0.000046062\cdot {\text{P}}^{2}-0.000056914\cdot {\text{C}}^{2}+0.003252103\cdot {\text{T}}^{2}\; & \; \end{array}$$

Where, Y_1_ or Z_1_ is log population reduction, *P* is Pressure in MPa, *C* is thymol concentration in ppm, *T* is pressure-holding time in minute, and *R*^2^ is 0.94 for Equation (2) and 0.98 for Equation (3). In Equation (2), all terms *P, C, T, PT, PC, CT, P*^2^, *C*^2^, and *T*^2^ were significant in the regression analysis with *P* < 0.05. However, in Equation (3) the interaction terms *PC and TC* were not significant in the regression analysis with *P* > 0.05, therefore, were not included.

**Figure 1:**
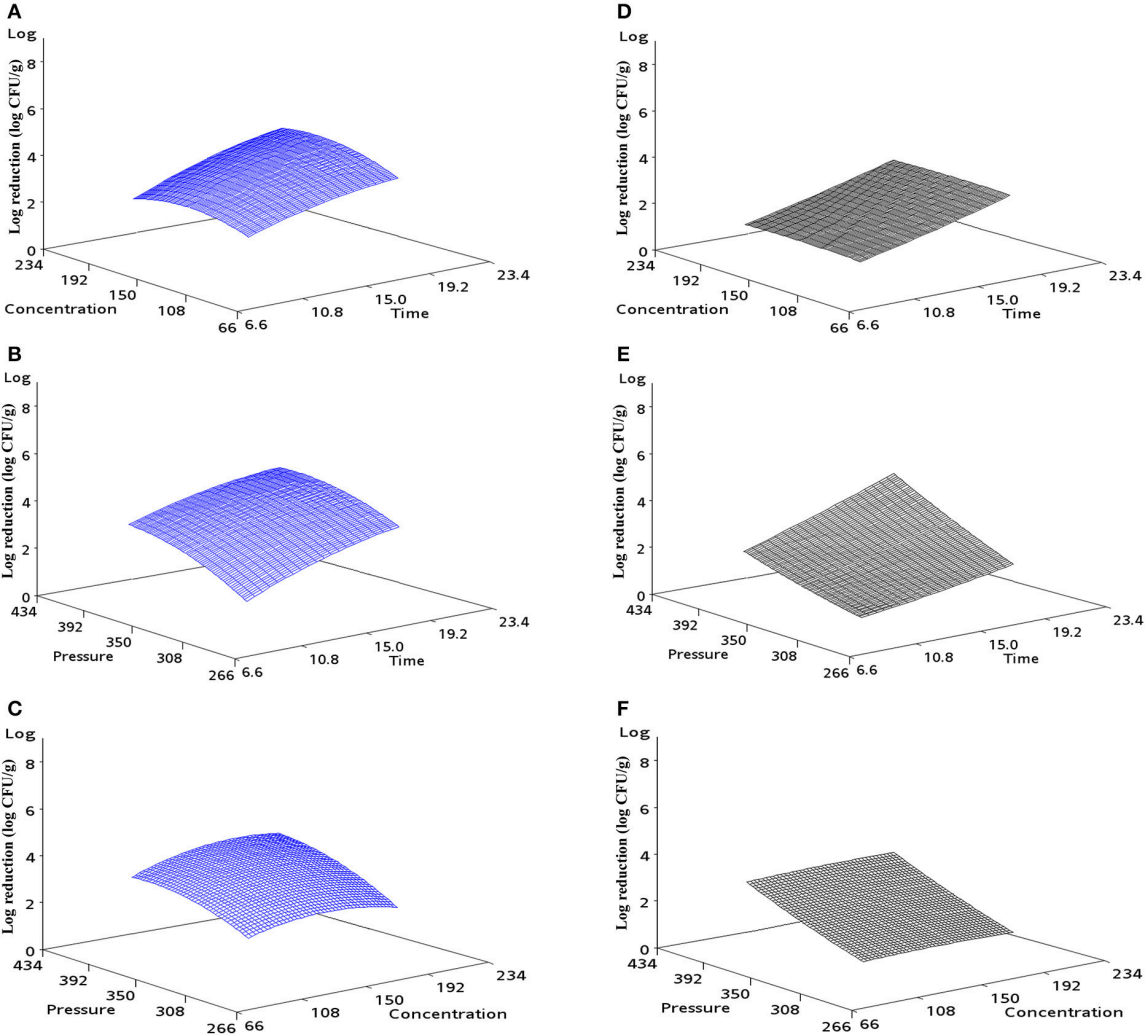
**Response surface 3D plot indicating the effect of (A) concentration and time, (B) pressure and time, and (C) pressure and concentration on iPEC O157:H7 on ground chicken**. Same as for UPEC with **(D–F)**.

The response surface curves can be used to explain the interaction of the variables and to determine the optimum level of each variable for a predicted maximum lethality. Figure 1 shows the predictive mathematical model visualized as a three-dimensional response plot. This plot shows when one variable is fixed at the coded 0 level: (a) pressure: 350 MPa; (b) thymol concentration: 150 ppm; (c) pressure-holding time: 15 min, how the remaining two variables (a): concentration and time; (b): pressure and time; (c): pressure and concentration interact with each other to affect reduction in the microbial population for either iPEC O157:H7 (a, b, c) or UPEC (d, e, f). All three selected parameters demonstrated their impact on the microbial log reductions of the pathogenic *E. coli* on ground chicken.

Canonical analysis confirmed that the critical values for those three factors may show a maximum point for iPEC O157:H7 (to attain 5 log CFU/g reduction), and the estimated optimum conditions and maximal response were the pressure at 397 MPa, pressure-holding time of 25 min, and thymol concentration at 196 ppm. Since canonical analysis of the surface response revealed that the stationary point for UPEC was at a saddle point, a ridge analysis was performed to determine the critical levels of the design variables that may have the maximum response. The estimated levels of each variable for maximum response (5 log CFU/g reduction) for UPEC were found: 413 MPa with 155 ppm thymol for 21 min. With a CCD design, which may involve the −α and +α parameter ranges (Table 2), those calculated optimal ranges may be considered acceptable in the model.

### Dimensionless non-linear model development for iPEC O157: H7 and UPEC

According to Zhou et al. (2015), Sheen's dimensionless non-linear model showed useful applications to simplify a model having multiple parameters. Therefore, his model was adopted to meet our purposes and achieved for the three factors, i.e., pressure, thymol concentration and pressure-holding time, model development. Those developed models are shown as Equations (4) and (5):

iPEC O157: H7:

(4) $$\begin{array}{r} {\text{Y}}_{2}=17.4960{\left[\frac{\text{P}-\;250.0}{\text{P}+250.0}\right]}^{0.4931}\;{\left[\frac{\text{C}-\;50.0}{\text{C}+50.0}\right]}^{0.3336}\\ {\left[\frac{T-\;6.0}{T+6.0}\right]}^{0.6454} \end{array}$$

UPEC:

(5) $$\begin{array}{r} {\text{Z}}_{2}=\;55.0025{\left[\frac{\text{P}-\;250.0}{\text{P}+\;250.0}\right]}^{1.1701}\;{\left[\frac{\text{C}-\;50.0}{\text{C}+50.0}\right]}^{0.1120}\\ {\left[\frac{T-\;6.0}{T+6.0}\right]}^{1.2357} \end{array}$$

**Figure 2:**
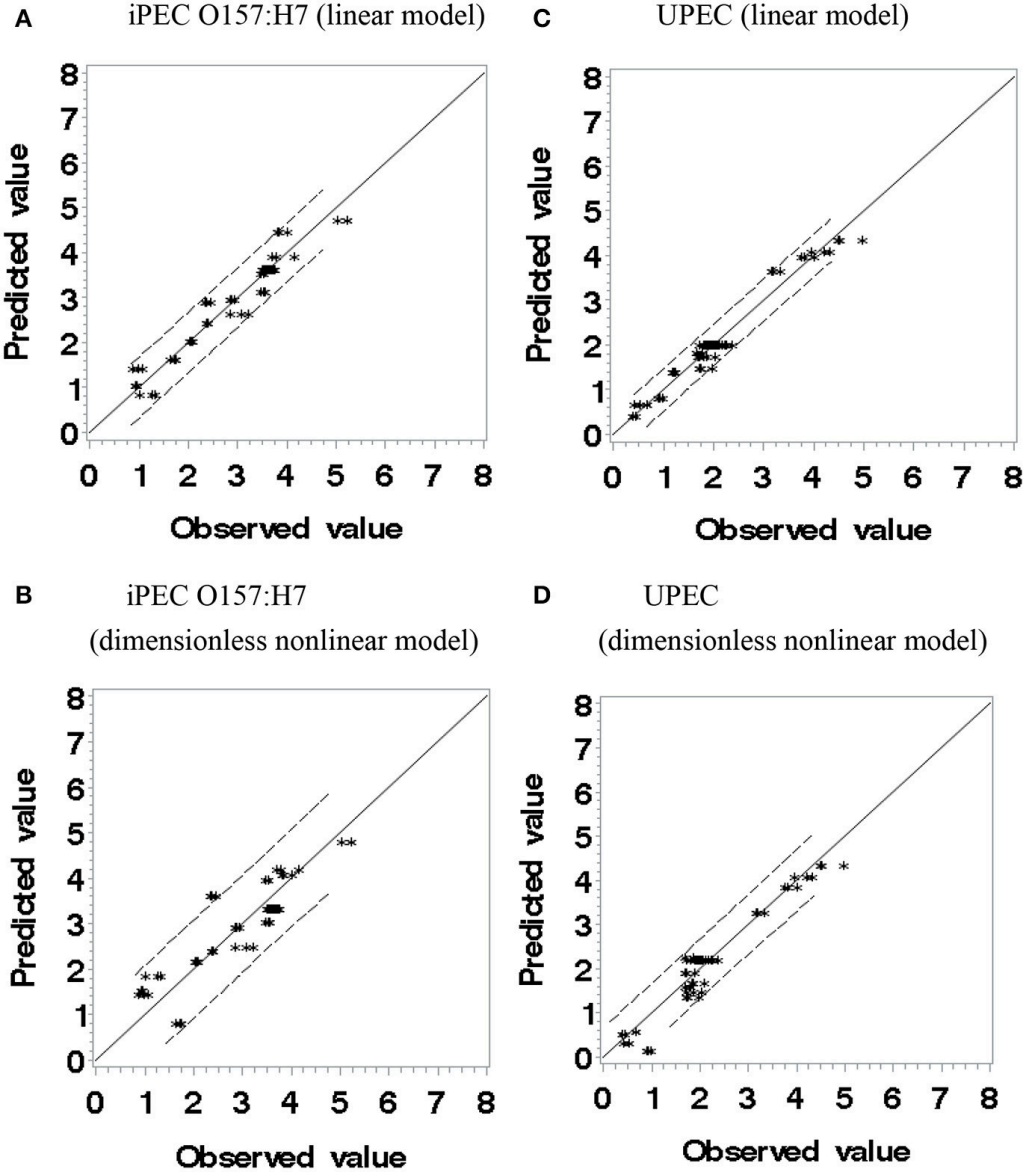
**The observed values vs. predicted values of log reduction (log CFU/g) using polynomial linear (A,C) and dimensionless non-linear models (B,D)**.

Where, 250.0, 50.0, and 6.0 are the pressure in MPa, thymol concentration in milligrams per liter and pressure-holding time in minute that reduction of iPEC O157: H7 and UPEC, selected from experimental observations. Those three numbers are at the lower end (minimal requirement) of each factor which may facilitate the regression procedure and application. The *F*-value was 542.35 and 761.42 for Equations (4) and (5), respectively; Pr > *F* (< 0.0001); and sum of squared error/uncorrected total is 15.1202/600.9 for Equation (4) and 5.9918/331.9 for Equation (5). The *F*-values and Pr > *F* values (used in non-linear regression) indicated the goodness of fit. Figure 2 shows observed values vs. predicted values using polynomial models and Sheen's dimensionless non-linear models. If the predicted values and observed values are equal, the data points should be on the solid lines (slope = 1.0). If the predicted values are over- or underestimated, the data points should be above or below the solid lines, respectively. From Figure 2, both types of models showed good fittings within 95% confidence limits. UPEC predicted with the linear polynomial model had a slightly narrower 95% confident range than iPEC O157:H7 one under the same conditions and have more data points located on or near the solid line (Figures 2A,C) indicating that the linear model for UPEC could be the best one in all. For application purpose, those models should perform equally well.

### Model validation

The experimental values were found in good agreement with the predicted values from Equation (2) to Equation (5). Model performance was validated using two additional experiment combinations, one HPP at 320 MPa and thymol concentration of 180 ppm for 17 min; and another one HPP at 390 MPa, and thymol concentration of 120 ppm for 14 min. All parameters were selected not on the CCD points but within the design parameter ranges. Table 4 presented the log reduction (experiment vs. prediction), all models showed good predictions (with derivation within 10%). The developed models were proven to be reasonably accurate for predicting the inactivation of iPEC O157:H7 and UPEC in ground chicken with treatment parameters in the range of 300–400 MPa, 100–200 ppm and 10–20 min. In addition, we further validate the dimensionless non-linear model with three factors outside design ranges, e.g., thymol concentration at 300 ppm, pressure at 425 MPa, and pressure-holding time at 30 min. The predicted values were 6.19 and 6.61 log reduction of iPEC O157:H7 and UPEC, respectively. The observed values were below detection limit (1.0 log CFU/g) indicating the reduction of iPEC O157:H7 and UPEC over 7 log CFU/g (or model slightly under estimated lethality).

**Table 4:** **Verification of predictive models (Equations 2–5) for log reduction of iPEC O157:H7 and UPEC in ground chicken**.

**Run**	**Parameter**			**Log**_**10**_ **reduction (CFU/g)^a^**					
	**Pressure (MPa)**	**Thymol conc. (ppm)**	**Time (min)**	**iPEC O157:H7**			**UPEC**		
				**Experiment**	**Predict (Equation 2)**	**Predict (Equation 4)**	**Experiment**	**Predict (Equation 3)**	**Predict (Equation 5)**
1	320	180	18	3.37 ± 0.25	3.53	3.29	1.80 ± 0.15	1.90	1.88
2	390	120	14	3.82 ± 0.06	3.48	3.40	2.22 ± 0.18	2.51	2.71
3	425	300	30	>7		6.19	>7		6.61

Initial populations of iPEC O157:H7 and UPEC on ground chicken were 9.02 ± 0.06 and 9.01 ± 0.08 log CFU/g, respectively. The detection limit was 1.0 log CFU/g.

a Values represent means ± standard deviations.

## Discussion

The resistance of iPEC O157:H7 and UPEC to HPP and thymol were compared. UPEC may be more resistant to high pressure treatment at 450 and 500 MPa. UPEC is becoming to be more troublesome foodborne pathogen in the food supply chain (Bélanger et al., 2011; Morran, 2013; Markland et al., 2015). This is the first systematic comparison report between STEC and UPEC in response to interventions (HPP alone and with an antimicrobial). UTIs exact a substantial public burden each year in terms of direct medical expenses, decreased quality of life, and lost productivity. According to several previous studies, UPEC may be transmitted from food animal sources and becoming of particular concern due to the strong indication that poultry may serve as a reservoir and supporting the hypothesis/evidence of urinary tract infections through the ingestion of contaminated food (Jakobsen et al., 2010; Vincent et al., 2010; Bergeron et al., 2012). UPEC may need to be treated as important as STEC as a foodborne safety concern.

Natural antimicrobial applications in food matrices to achieve foodborne pathogen reduction or lethality should be used carefully especially hurdled with other processing options. The thermal effect may destroy the effective compounds. The complexity of foods in the consumer products may have detrimental impact on certain components. The natural antimicrobials could be a mixture of many components and some were not identified yet. Lucera et al. (2012) discussed and reviewed the food applications using natural antimicrobial compounds where many were involved in shelf life extension. Perricone et al. (2015) reviewed the bioactivity of essential oils and their interactions with proteins, carbohydrates, oils, and etc. Calo et al. (2015) reviewed the essential oils (EOs) as antimicrobials in food systems and concluded that many EOs exhibited activity against foodborne pathogens and spoilage organisms *in vitro* and to a small degree in foods. We were aware that in the HPP application, some natural antimicrobials showed little function and some needed much higher dose to perform the meaningful lethality in real foods compared with the results in culture medium. Therefore, the true effectiveness of EOs in real food applications should be verified on case by case basis.

It is also interesting to know that HPP alone did not show significant inactivation difference between iPEC O157:H7 and UPEC (*p* > 0.05) at the pressure below 400 MPa (Table 2). However, Table 3 demonstrated that there was significant difference (each pair of trial number) on lethality between iPEC O157:H7 and UPEC (*p* < 0.05), except pair #9 (trial #9) with the pressure at 434 MPa. UPEC may become more resistant than iPEC O157:H7 based on the observations in Table 3. HPP coupled with thymol (or other antimicrobials) could impose stress complexity on microbes to react differently. This phenomenon may need to be further investigated.

Response surface methodology (RSM) is a collection of mathematical and statistical techniques for empirical modeling, where a response of interest is influenced by several variables considered and the objective is to optimize this response (Montgomery, 2005). Zhu et al. (2010) used RSM to determine the optimum levels of different variables to optimize the microwave-assisted extraction of astaxanthin from Phaffia rhodozym, which provides some fundamental information for applying RSM to optimize food processing. According to the best fitting polynomial equation, pressure, holding time and thymol concentration were the important factors determining the extent of HPP inactivation of iPEC O157:H7 and UPEC on ground chicken. The main linear terms of pressure, holding time and thymol concentration were included in the model, indicating that treatment efficiency improved as level of those three factor increased. Thymol may show strong antibacterial function against foodborne pathogens such as *S. typhimurium, Listeria monocytogenes*, and *E. coli* (Burt, 2004) but largely depending on the food matrix involved. The inactivation of iPEC O157:H7 and UPEC on ground chicken with thymol concentration impact were demonstrated in this study. It is difficult to disperse thymol evenly in food matrices when a dose above the solubility is required (Pan et al., 2014). The chicken meat is a fairly good matrix to have thymol (pre-dissolved in ethanol) mixed in. NACMCF requires a 5-log CFU/g reduction of pathogenic *E. coli* (i.e., iPEC) strains in ground poultry using pasteurization technology including HPP. In this research, at 500 MPa for 15 min 7.20 and 5.23 log reductions were obtained for the iPEC O157:H7 and UPEC, respectively. However, texture may be damaged at pressures >400 MPa. By contrast, at 400 MPa with 200 ppm thymol for 20 min 5.16 and 4.66 log reductions were obtained for the iPEC O157:H7 and UPEC, respectively. The cost of HPP operation is related to high pressure and holding time, so it is necessary to achieve the acceptable operation cost with food quality and microbial safety concerns (Bover-Cid et al., 2011).

A multiple-factor model development is typical time-consuming due to its complexity in experimental design. However, several models taking into account of pressure, antimicrobial agent, and holding time are unique to its application in ground chicken with iPEC O157:H7 and UPEC contamination were constructed. According to the principle of dimensionless non-linear model, extra terms can be added to generate the general multiple-factor model which can be expressed as Equation (6).

(6) $$\begin{array}{l} \text{Reduction or Lethality},\;\text{L}=\text{k}\prod_{\text{i}=1}^{\text{n}}{\left[\frac{{\text{X}}_{\text{i}}-{\text{X}}_{\text{min/or max}}}{{\text{X}}_{\text{i}}+{\text{X}}_{\text{min/or max}}}\right]}^{\text{mi}} \end{array}$$

Where *k* and *m*_*i*_ is the constant or the exponent to be determined, *X*_*i*_ may be any parameter and *X*_*min*∕*or max*_ is a level with its minimum (or maximum) impact on bacteria.

Generally speaking the more factors involved, the higher difficulty incurred in the model development. This research developed and validated two different types of regression models, i.e., polynomial linear model and the dimensionless non-linear model to predict the inactivation (lethality) of iPEC O157:H7 and UPEC in the ground chicken as impacted by thymol, pressure, and holding time. The dimensionless non-linear model may be applied to the parameter ranges slightly outside the CCD limits which broader its application to attain the lethality >5.0 log CFU/g. For example, an increase of process time to achieve the >5 log CFU/g reduction can be predicted using the dimensionless non-linear models (Equations 4, 5). The availability of predictive models describing iPEC O157:H7 and UPEC inactivation may provide sound scientific data for practical applications to the food industry to reduce microbial safety risks.

## Conclusion

iPEC O157:H7 and UPEC were compared for their resistance to high hydrostatic pressure stress and thymol essential oil. HPP technology alone or in combination with antimicrobials was proved a feasible means to enhance food safety. Although, analytical models in which physical parameters directly used to describe the inactivation may provide better understanding in cell survival, they are very difficult to develop due to the complexity of food system and mathematics involved. Regression models with proper experimental design are relatively easy to attain. Our regression models (both linear and dimensionless non-linear) may be used to assist government and food industry in the risk assessment task. In addition to thymol, other potential food-grade antimicrobials may be available for the real food applications to significantly reduce foodborne pathogens which remain to be further explored and validated.

## Author contributions

SC Candidate who has completed the experiment and data analyses and write-up. SS Co-advisor who provided lab and advices to student, manuscript write-up and submission process. CS Scientist provided information and strains of uropathogenic *E. coli* and feedbacks. LS Co-advisor at National Taiwan University with general advices to the study.

## Funding

This Project was funded by USDA-ARS National Program 108 Food Safety Project No. 8072-42000-073-00D.

## Disclaimer

Mention of trade names or commercial products in this publication is solely for the purpose of providing specific information and does not imply recommendation or endorsement by the U.S. Department of Agriculture. USDA is an equal opportunity provider and employer.

### Conflict of interest statement

The authors declare that the research was conducted in the absence of any commercial or financial relationships that could be construed as a potential conflict of interest.
